# The Inhibitory Effects of Npas4 on Seizures in Pilocarpine-Induced Epileptic Rats

**DOI:** 10.1371/journal.pone.0115801

**Published:** 2014-12-23

**Authors:** Dan Wang, Min Ren, Jiamei Guo, Guang Yang, Xianghua Long, Rong Hu, Wenjing Shen, Xuefeng Wang, Kebin Zeng

**Affiliations:** 1 Department of Neurology, the First Affiliated Hospital of Chongqing Medical University, Chongqing Key Laboratory of Neurology, Chongqing, China; 2 Department of Psychiatry, the First Affiliated Hospital of Chongqing Medical University, Chongqing, China; Université Pierre et Marie Curie, France

## Abstract

To explore the effects of neuronal Per-Arnt-Sim domain protein 4 (Npas4) on seizures in pilocarpine-induced epileptic rats, Npas4 expression was detected by double-label immunofluorescence, immunohistochemistry, and Western blotting in the brains of pilocarpine-induced epileptic model rats at 6 h, 24 h, 72 h, 7 d, 14 d, 30 d, and 60 d after status epilepticus. Npas4 was localized primarily in the nucleus and in the cytoplasm of neurons. The Npas4 protein levels increased in the acute phase of seizures (between 6 h and 72 h) and decreased in the chronic phases (between 7 d and 60 d) in the rat model. Npas4 *expression* was knocked down *by* specific *siRNA interference*. Then, the animals were treated with pilocarpine, and the effects on seizures were *evaluated on the 7th day*. The onset latencies of pilocarpine-induced seizures were decreased, while the seizure frequency, duration and attack rate increased in these rats. Our study indicates that Npas4 inhibits seizure attacks in pilocarpine-induced epileptic rats.

## Introduction

Epilepsy often occurs in patients suffering from recurrent seizures, which are usually associated with an imbalance of excitatory and inhibitory neurons in the central nervous system (CNS), as demonstrated by Brenner [4]. Epilepsy is usually associated with neuronal loss [8], astrocyte proliferation [27], mossy fiber sprouting [31], and synaptic reorganization in the hippocampus. Epilepsy is known to be resulted from an imbalance between glutamate-mediated excitatory and GABAergic inhibitory networks [1], [2], which are closely related to ionotropic receptor composition and function, glutamate or GABA transporters function, calcium-mediated second messenger activity, intrinsic properties of neuronal membranes, endogenous anticonvulsant and neuroprotective activities [2], [27]. In the mammalian brain, an imbalance in neural network damage has been highly associated with neurological and neurodegenerative diseases, such as epilepsy [32], Parkinson’s disease [16], and Huntington’s disease [26].

Neuronal Per-Arnt-Sim domain protein 4 (Npas4), also known as neuronal transcription factor Nxf, and limbic-enriched PAS domain protein (LE-PAS) belongs to the basic helix-loop-helix family, which has been classified into Npas1, Npas2, Npas3, and Npas4 [5], [21], [38]. Npas4 is specifically expressed in the central nervous system (CNS), which is predominantly restricted to the cortical and hippocampus areas of the rodent brain [21], [25]. Npas4 is an important transcriptional regulator that plays critical roles in developmental, physiological and pathological events. Npas4 adjusts the homeostatic mechanisms of inhibitory and excitatory neurons. Additionally, Npas4 has been identified as a brain-specific transcription factor and may serve a neuroprotective function [20]. Npas4 has been demonstrated to critically regulate inhibitory synapse formation [15] and to directly control brain-derived neurotrophic factor (BDNF) activity-dependent transcription [24]. Npas4 plays a role in the development of inhibitory synapses by regulating the expression of activity-dependent genes, which in turn control the number of GABA-releasing synapses that form on excitatory neurons [15]. Npas4 is also required for the activity-induced changes in synaptic inputs to these neurons but not for changes to output synapses in their axons [30].

However, it is still unknown whether Npas4 participates in the pathogenesis of epilepsy. Although several studies have suggested that Npas4 may be related to seizures and psychiatric disorders [1], [9], [15], the exact mechanism by which Npas4 participates in the inhibitory pathway and leads to the symptomatology of developmental disorders, such as epilepsy, has not been fully determined. To explore the effects of Npas4 on seizures in pilocarpine-induced epileptic rats, we have detected the dynamic expression of Npas4 in the hippocampus and adjacent cortex using double-label immunofluorescence, immunohistochemistry, and Western blotting. We have further explored the effects of Npas4 seizures on epileptic rats after down-regulating the Npas4 *expression by* specific *siRNA interference*.

## Materials and Methods

### Pilocarpine-Induced Epilepsy

Healthy young male Sprague Dawley rats (weighing 200–220 g) were obtained from the Experimental Animal Center of Chongqing Medical University. The rats were housed in cages and maintained suitable conditions (ambient temperature 24–25°C, humidity 50–60%, 12 h light and 12 h dark cycle, free access to standard food and water). The experimental procedures were approved by the Commission of Chongqing Medical University for ethics of experiments on animals and were conducted in accordance with international standards. The pilocarpine model reproduces most of the clinical and neuropathological features of epilepsy and has been widely used for the study [17], [23], [34], [35]. The rats were divided into a control group and six subgroups (n = 8 per subgroup) stratified by the period after status epilepticus: 6 h, 24 h, 72 h, 7 d, 30 d, and 60 d, respectively. The experimental rats were injected intraperitoneally with lithium chloride (127 mg/kg, i.p., Sigma-Aldrich, Media Cybe-metrics, Pierce) 14–18 h before the first pilocarpine administration (50 mg/kg, i.p., Sigma Aldrich, USA). Pilocarpine (10 mg/kg, intraperitoneally) was given repeatedly every 30 min until the rats developed seizures. The total number of pilocarpine injections was limited to five injections per rat. Seizures were scored in each rat using Racine’s scale, which was determined by behavioral assessment considering only convulsive (motor) seizures for scoring: stage 4, rearing; stage 5, rearing plus loss of balance and falling that was accompanied by generalize clonic seizures. Sixty minutes after the onset of the status epileptics, the animals were injected with diazepam (10 mg/kg, intraperitoneally) to terminate seizures. Rats in the control group were injected with normal saline instead of pilocarpine. All rats were video-monitored (8 hours/day, 5 days/week) to record spontaneous seizures after pilocarpine injection. Animals were sacrificed 6 h, 24 h, 72 h, 7 d, 30 d, and 60 d after status epilepticus. Both the hippocampus and adjacent cortex were removed.

### Lentivirus Production and Stereotactic Injection

Npas4 siRNA was synthesized by GeneChem (Shanghai, PR.China). The coding sequence of shRNA for Npas4 was amplified by reverse transcription polymerase chain reaction and ligated them into the pGC-FU plasmid to produce pGC-FU-Npas4-green fluorescent protein (GFP) (LV-Npas4-sh). A lentiviral vector expressing GFP alone (LV-GFP) was chosen as a control. Different shRNAs targeting Npas4 gene were screened in primary culture neurons. The LV-Npas4-sh that effectively knocked down the expression of Npas4 was chosen. The Npas4 -specific siRNA was designed as the report [15], 5′-GGTTGACCCTGATAATTTA-3′. The titer of the lentivirus (LV) was 2×10^9^ Tu/mL(Shanghai GeneChem Corporation, PR.China).

Stereotaxic intra hippocampus injection was done as the report [12]. Twenty-four male rats were deeply anesthetized by intraperitoneal injections of 3.5% chloral hydrate (1 mL/100 g), and the rat’s head was fixed in a stereotaxia frame (Stoelting Co. Ltd., USA). A volume of 5 µL LV-Npas4-sh (LV-Npas4-sh group, n = 8) and LV-GFP (LV-GFP group, n = 8) were infused through a glass pipette (0.2 µL/min) bilaterally in the dorsal hippocampus (anterior –posterior −3.3 mm, medial–lateral ±1.8 mm, and dorsal–ventral −2.6 mm). The pipette was placed at least 5 min after injection to prevent back flow. In the normal saline control group (n = 8), the lentiviral vector was replaced by equal volume of normal saline, and the other 8 rats with no treatment as the normal control group. After injection of Lentivirus, all of animals were reared in a standard environment for 7 d, and then given pilocarpine (40 mg/kg) to observe acute seizure features for 2 h.

### Tissue Processing

At every time points after the initiation of seizure, four rats of each group were sacrificed by decapitation after intraperitoneally injection chloral hydrate (1 ml/kg). The temporal lobes were dissected and stored in liquid nitrogen for western blot analysis. The remaining rats were anesthetized and intracardially perfused with 0.9% saline followed by 4.0% paraformaldehyde. And then, brain tissues were removed. After being fixed in formalin for 48 h, they were embedded regularly with paraffin. Serial sections (10 µm thick) were taken and 10 consecutive sections for each case, then five sections from each case were randomly selected for staining.

### Immunohistochemistry

Paraffin sections were used for Npas4 immunohistchemistry staining, which were performed according to the standard avidin-biotin peroxidase complex protocol. Briefly, tissue sections were deparaffinated in xylene for 30 min, then rehydrated in a graded series of ethanol (100, 95, 80, and 70%) with 5 min for each grade, and incubated in 0.3% H_2_O_2_ for 10 min and antigen was recovered by use of microwave. Sections were heated (92–98°C) in 10 mmol/L boiling sodium citrate buffer at pH 6.0 for 20 min. Then washed with 0.1 M phosphate buffered saline (PBS), pH 7.4, and blocked for 30 min in 10% normal goat serum (Zhongshan Golden Bridge, Beijing, China) at room temperature. Sections were then overnight incubation with the primary anti-NPAS4 antibody (rabbit polyclonal antibody, absorption1∶100, Novus biological, USA) at 4°C. Next, the sections were washed with PBS for 5 min and incubated with secondary goat anti-rabbit (1∶100) at 37°C for 30 min, Sections were then treated with ABC solution (Zhongshan Golden Bridge Inc., Beijing, China) for 30 min, washed in PBS, and then stained with 3, 3′-dia-minobenzidine tetrahydrochloride (DAB, Zhongshan Golden Bridge) for 3 min. Counterstaining was performed using Harris’s hematoxylin. For negative controls, sections were also incubated in PBS in the absence of primary antibodies. We obtained 10 visual field images from every section using an OLYMPUS PM20 automatic microscope (Olympus, Tokyo, Japan) and a TCFY-2050 pathology system (Yuancheng, Beijing, China). Using the Motic Med 6.0 CMIAS pathology image analysis system (Beihang Motic, Beijing, China), the mean percentages of Npas4-positive cells in each visual field images were calculated for each section.

### Double Immunofluorescence Labeling

The rat cryosections were thawed at room temperature and then fixed in acetone solution for 30 min before staining. After several washes in PBS, the sections were preincubated with 0.4% Triton for 30 min, washed in PBS, and blocked with normal goat serum (Zhongshan Golden Bridge, Beijing, China) at room temperature for 1 h. For double labeling, the tissue sections were incubated with a mixture of anti-Npas4 antibody (1∶100, Novus biological, USA) and anti-MAP2 antibody (1∶100, mouse monoclnal antibody, Boster, Wuhan, China) or mouse anti-GFAP antibody (1∶100, Zhongshan Golden Bridge, Beijing, China) at 4°C overnight. Tissue sections were washed with PBS and incubated with fluorescein isothiocyanate (FITC)-conjugated goat anti-rabbit IgG (1∶50; Zhongshan Golden Bridge, Beijing, China) and Tetramethyl rhodamine isothiocyanate(TRITC)-conjugated goat anti-mouse IgG (1∶50; Zhongshan Golden Bridge, Beijing, China) in the dark room for 90 min at 37°C, then washed with PBS three times for 10 min each, and mounted in 1∶1 glycerol/PBS. Fluorescent images were collected using laser scanning Confocal microscopy (Leica Microsys-tems Heidelberg GmbH, Germany) on an Olympus IX 70 inverted microscope (Olympus, Japan) equipped with a Fluoview FVX confocal scan head.

### Western blotting analyses

Western blot analysis was performed as described in previous reports [15], [37]. Homogenates obtained from animal brain tissues were frozen in liquid nitrogen. A whole protein extraction kit (Keygen biotech, Nanjing, China) was used to extract proteins, and an enhanced BCA Protein Assay Kit (Beyotime, Haimen, China) was used to determine the protein concentrations. A total protein of 50 µg was laded in each land and then separated with SDS-PAGE (5% spacer gel, 80 V, 30 min; 10% separating gel, 100 V, 60 min) and then electrotransferred onto immobilon polyvinylidene difluoride membranes (PVDF, Millipore Corporation) at 250 mA for 120 min with an electrophoretic transfer system (Bio-Rad Laboratories). Equivalent protein loading and transfer were confirmed by Ponceau S staining of the membranes. The PVDF membrane was incubated at 37°C for 2 h in 5% BSA to block nonspecific binding and then incubated with monoclonal rabbit anti-Npas4 (1∶500, Novus Biotechnology, USA) at 4°C overnight. After extensive rinsing with TBST (Tween-20-Tris-buffered saline) four times (10 min per time), the membranes were incubated with a horseradish peroxidase-conjugated secondary antibody (1∶5000; goat anti rabbit IgG-HRP Zhongshan Golden Bridge, China) for 1 h at 37°C and then washed as previously described. Anti-β-actin (1∶5000, Zhongshan Golden Bridge) labeling was used to normalize for protein loading. The resulting protein bands were visualized by an enhanced chemiluminescence substrate kit (Beyotime institute of Biotechnology, China) and digital scanned (Bio-Rad Laboratories). The image pixel density was quantified using the Quantity One software (Bio-Rad Laboratories). Band immune intensity ratio of Npas4 and corresponding β-actin at the same time of electrophoresis were analyzed. The band intensity ratio of Npas4 relative to β-actin (Npas4/β-actin) was analyzed.

### Statistical analysis

All values were presented as the mean ± SD. Student’s t test was used for statistical analysis of the differences between the control and each group of experimental rats. Differences among groups of experimental rats were determined by One-way ANOVA analysis followed by Tukey’s HSD posthoc multiple comparison test (SPSS 17.0). Values of *p*<0.05 were considered statistically significant.

## Results

### Investigation of Animal Behavior

In pilocarpine-induced temporal epilepsy rats, pilocarpine injection induced the onset of continuous seizures in approximately 10–60 minutes. However, the onset latency differed between the rats. During seizures, typical manifestations were observed, including chewing, trembling, salivation, continuous scratching, clonic movements of fore-limbs, rearing, strong body tremor and falling. Only the rats that exhibited convulsive seizures were used for further analysis. Most rats were kindled, except for the following three groups: the 6 h, 72 h and 30 d groups. Seizures were stopped by a diazepam injection (10 mg/kg, intraperitoneally) 60 min after initiating the seizures. The rats were then monitored for different periods, according to their groups (i.e., 6 h, 24 h, 72 h, 7 d, 14 d, 30 d, and 60 d). The frequency of secondary episodes differed among the animals; some animals remained seizure-free for many days, while the others had frequent seizures. The frequency of seizure episodes in the acute and chronic phases was greater than in the latency phase. Four rats died after kindling, one in the 3 d group, one in the 30 d group, and two in the 60 d group. There were no behavioral changes found in the normal control group rats.

### Double-label Immunofluorescence

**Figure 1:**
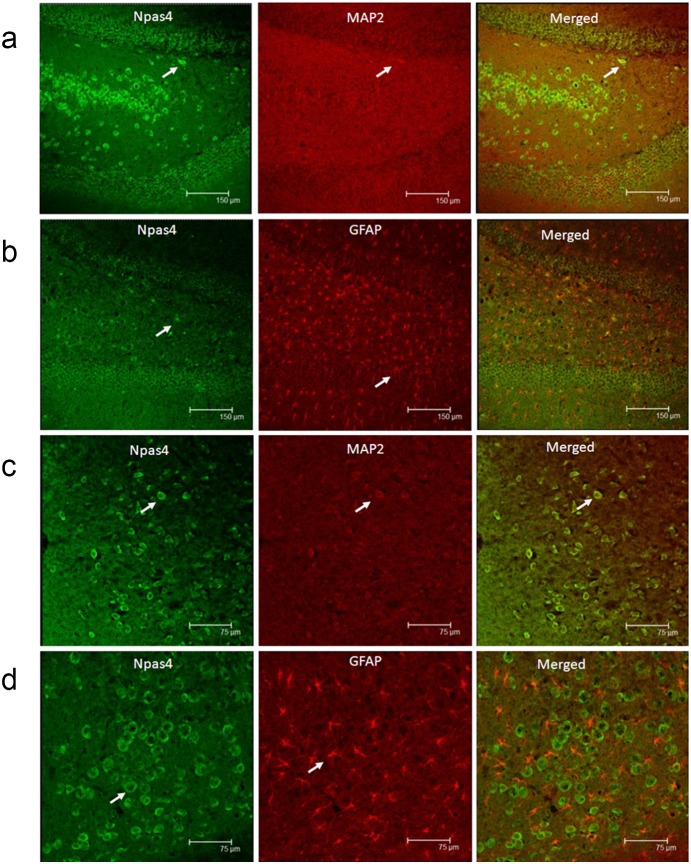
A confocal microscopic image of Npas4 in different rat brain tissues. Npas4 (green) and MAP2 (red) are co-expressed within neurons in the rat hippocampus and neocortex (a, c). Npas4 (green) and GFAP (red) are not co-expressed in the astrocytes in the hippocampus and cortex (b, d). Scale bar: (a, c) 150 µm, (b, d) 75 µm.

In the epilepsy rat model, double-label immunofluorescence staining showed that Npas4 and MAP2 were co-expressed in neurons in the cortex and hippocampus (Fig. 1.a.c) but were not co-expressed with GFAP, an astrocyte marker, in neuroglia cells in the hippocampus and cortex (Fig. 1.b.d).

### Immunohistochemistry

**Figure 2:**
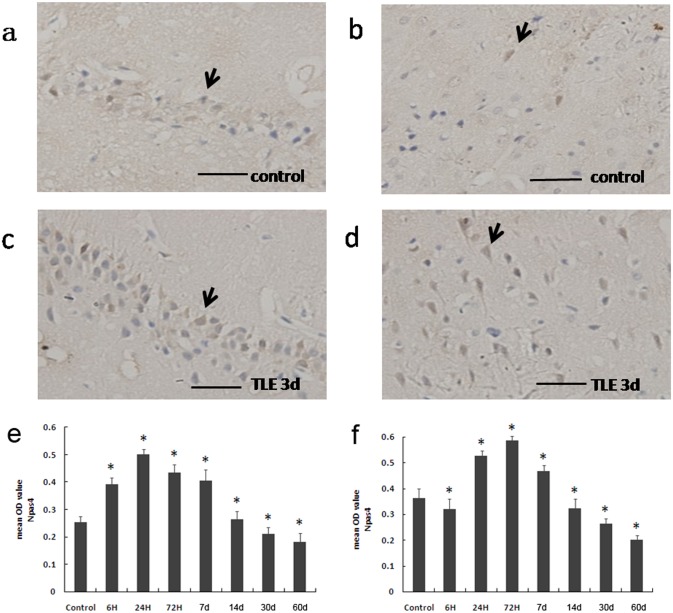
Immunohistochemistry showed immunoreactive staining of Npas4 in the hippocampi (a) and adjacent cortices (b) of the normal control rat brains. Immunohistochemistry of Npas4 showed strong immunoreactive staining in the hippocampus (c) and adjacent cortex (d) at 72 h after kindling. The black arrowheads indicate Npas4-positive cells. (e) A comparison of the mean OD value between the epileptic and normal rats. The mean OD value indicated that Npas4 expression in the hippocampi of the rats increased at 6 h after the pilocarpine-induced seizure. The highest level of Npas4 expression occurred at 24 h and then began to decline, reaching its lowest level at 30 d. The Npas4 expression levels differed significantly between normal and epileptic rats between 6 h and 30 d after kindling (*P<0.05). (f) A comparison of the mean OD values between epileptic and normal rats. The mean OD value indicated that Npas4 expression in the adjacent cortices of the rats increased at 24 h after pilocarpine-induced seizure and reached its highest level at 72 h. The lowest level of Npas4 expression occurred at 60 d. There were significant differences in Npas4 expression levels between the normal and epileptic rats from 6 h to 60 d after kindling (*P<0.05).

Npas4-positive cells were examined in the hippocampi and adjacent neocortices of rats in the control and epileptic groups. Npas4-positive cells were mainly located in the nuclei of neurons in the hippocampus and cortex (Fig. 2.a.c.). Most Npas4 expression was observed in the dentate gyrus, CA1 and CA3 regions. No staining was observed when the primary antibody was omitted.

The mean OD value of Npas4 expression changed dynamically during different phases (Fig. 2.e.f.) as indicated by semi-quantitative immunohistochemistry. Npas4 expression in the rat hippocampus gradually increased during the acute period (i.e., 6 h, 24 h and 72 h) and peaked at 24 h before decreasing below the levels observed in the controls during the chronic phase (30 d and 60 d); the lowest level was observed at 60 d (Fig. 2.e). Compared to the control group, Npas4 expression in the adjacent neocortex was increased in the acute phase (24 h, 72 h, 7 d) in the epileptic groups and was decreased in the 14 d, 30 d and 60 d epileptic groups (*P<0.05) (Fig. 2.f.).

### Western blot analysis

**Figure 3:**
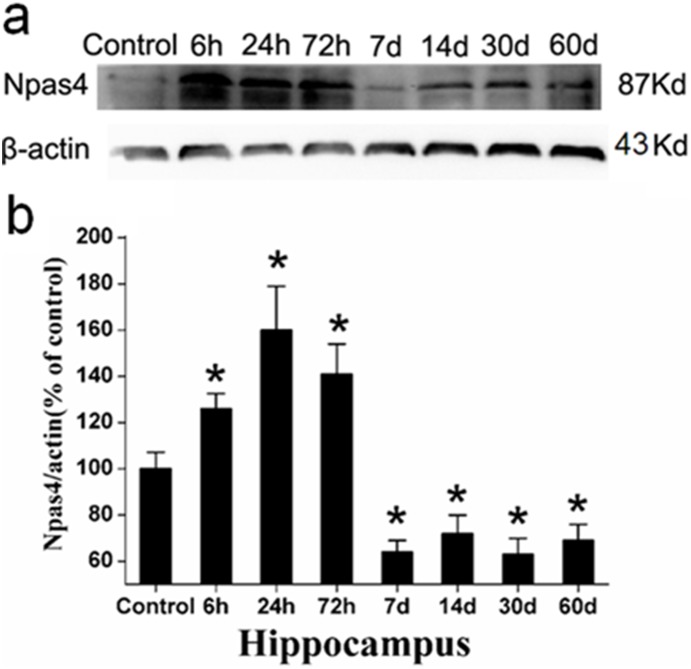
Western blot analysis of Npas4 expression in the hippocampi of pilocarpine-induced epilepsy rats. (a) Npas4 expression (band at 87 kDa) at different time points in the treatment and control rats. β-actin (band at 43 kDa) was used as a positive control. (b) Quantitative analysis of the mean OD ratio of Npas4 in the rat hippocampi. *P<0.05 was considered to indicate a significant difference between the controls and the different time-point groups.

Western blot analysis was used to further analyze Npas4 protein. Npas4 presented as a band of protein at approximately 87 kDa (Fig. 3.a). In the hippocampus, the Npas4 protein expression began to increase at 6 h, peaked at 24 h and was transiently increased at 72 h after the seizure compared with the control group. However, Npas4 expression then decreased at 14 d and significantly decreased at 30 d and 60 d (Fig. 3.b). The lowest Npas4 expression level was at 7 d. The difference in the Npas4 expression levels between the control group and the treatment groups was significant (*P<0.05). Consistent with the immunohistochemistry results, the Npas4 expression levels commonly peaked at 24 h, and both analyses suggested that the expression levels increased in the acute phase and decreased in the chronic phase compared to the control groups. Although there are subtle differences between the results, the overall trend was consistent.

### An analysis of Npas4 expression after siRNA interference

**Figure 4:**
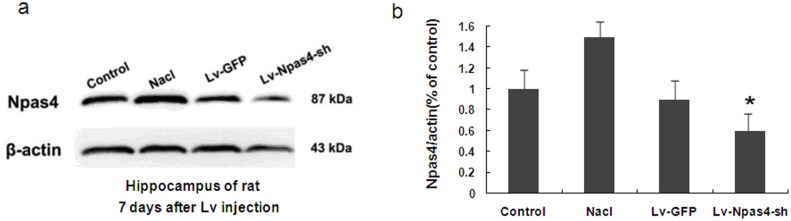
Npas4 protein expression 7 d after the lentivirus injection. (a) Npas4 expression (band at 87 kDa) in different groups after induction of seizures. β-actin (band at 43 kDa) was used as a positive control. (b) Quantitative analysis indicated that the mean OD ratio of Npas4 expression in the rat hippocampi decreased significantly in the Lv-Npas4-sh group (*P<0.05).

Using Western blot analysis, Npas4 protein expression was detected at 7 d after the siRNA intervention. Npas4 protein expression was significantly decreased in the Lv-Npas4-sh group compared with the normal, saline, and empty vector groups (P<0.05) (Fig. 4).

### Kindling effects after injecting lentivirus

**Figure 5:**
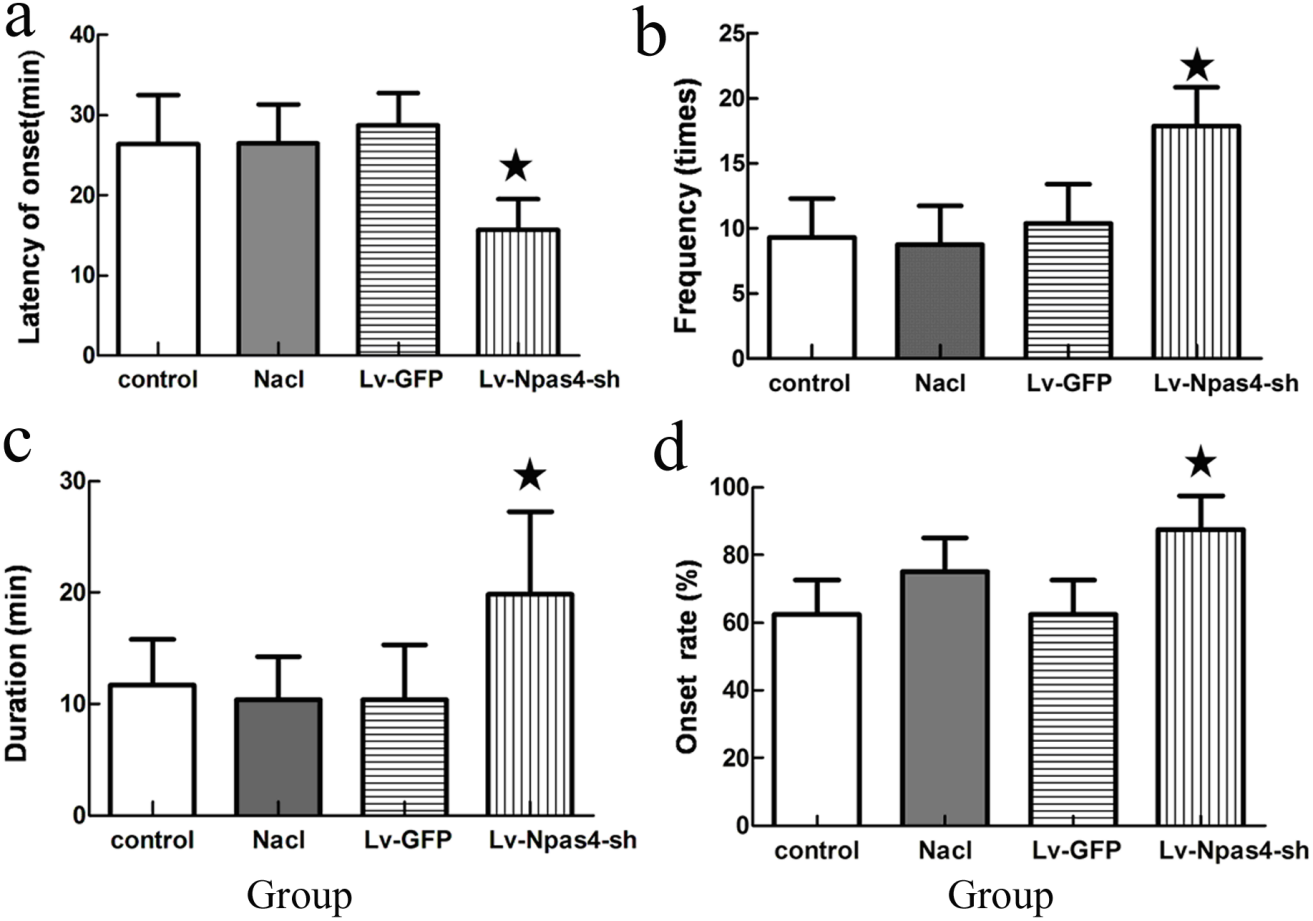
Seizure changes in each group within 2 h after injecting the pilocarpine. (a) The onset latency in the Lv-Npas4-sh group was significantly shorter compared to the other three control groups (*P<0.05). (b) The number of attacks in the Lv-Npas4-sh group was significantly greater compared to the other three groups (*P<0.05). (c) The duration in the Lv-Npas4-sh group was significantly increased compared with the durations in the three control groups (*P<0.05). (d) The attack rate in the Lv-Npas4-sh group was significantly greater than the attack rates in the three control groups (*P<0.05).

After injecting pilocarpine intraperitoneally (40 mg/kg), the seizure onset latencies were 26.40±6.10 min in the normal control group, 26.50± 4.89 min in the saline control group, 28.80±3.96 min in the empty vector group and 15.71±3.86 min in the Lv-Npas4-sh group. Onset latency was significantly reduced in the Lv-Npas4-sh group compared to the other three control groups (P<0.05), while there were no significant differences in onset latency between the three control groups (P>0.05) (Fig. 5.a).

Within 2 h after the pilocarpine injection, the mean rank seizure frequency was 9.30 times in the normal group, 8.75 times in the saline group, 10.40 times in the empty vector group, and 17.96 times in the Lv-Npas4-sh group. The number of attacks in the Lv-Npas4-sh group was significantly greater than those in the other three groups (P<0.05) (Fig. 5.b).

The mean seizure duration was 11.67±4.13 min in the normal group, 10.40±3.85 min in the saline group, 10.40±4.88 min in the empty vector group and 19.86±7.43 min in the Lv-Npas4-sh group. There was no significant difference between the control groups (P>0.05); however, the seizure duration in the Lv-Npas4-sh group was significantly greater compared to the three control groups (*P<0.05) (Fig. 5.c).

The seizure onset rate after pilocarpine injection was 62.5% in the normal group, 75% in the saline group, 62.5% in the empty vector group and 87.5% in the Lv-Npas4-sh group. The seizure onset rate in the Lv-Npas4-sh group was significantly greater compared to the seizure onset rate in the three control groups (P<0.05) (Fig. 5.d).

## Discussion

Npas4 expression was dynamically detected in a pilocarpine-induced epileptic rat model. The effects of Npas4 on seizures were explored after the down-regulation of the Npas4 expression using siRNA interference. The main results in our study were as follows: (1) in pilocarpine-induced epileptic rats, the expression of Npas4 was significantly increased during the acute phase but decreased during the recovery and chronic phases. These dynamic changes indicated that Npas4 is strongly related to seizure attacks. Npas4 may play an important role in the pathogenesis of epilepsy. (2) After the down-regulation of Npas4 gene expression by specific siRNA interference, the seizure frequency, duration and attack rates were significantly increased, while the onset latencies of seizures were significantly decreased, compared to the control groups. The results demonstrated that Npas4 inhibits seizures in pilocarpine-induced epileptic rats.

In our study, Npas4 protein expression was investigated in the hippocampi and adjacent neocortices of pilocarpine-induced epilepsy rats. Immunohistochemistry indicated that Npas4 expression was significantly increased in the hippocampus during the acute phase (6 h, 24 h) after epileptic seizures in pilocarpine-treated rats and then decreased during the recovery phase (3 d, 7 d, 14 d) and chronic phase (30 d, 60 d); these results were confirmed by using Western blot analysis. Under normal conditions, Npas4 mRNA is expressed at low levels, if at all, but it can be rapidly and robustly up-regulated in response to various stimuli. The primary signal for inducing Npas4 expression is an increased concentration of nuclear calcium (Ca^2+^), which is largely modulated by excitatory neural activity in neurons. Other conditions that induce the up-regulation of Npas4 mRNA include signaling through neurotrophin receptors, various drugs of abuse and some forms of cellular stress. In each case, the Npas4 induction is extremely rapid, with both mRNA and protein expression reaching maximum levels within 30–90 minutes of stimulation. However, this up-regulation is generally transient, and once peak expression is reached, both mRNA and protein levels decline sharply. Thus, Npas4 expression returns to baseline levels within a few hours. This type of rapid-response expression pattern is characteristic of a group of genes known as ‘immediate-early genes’ (IEGs). The dynamic changes in Npas4 expression suggest that the gene may be regulated in a similar manner to IEGs [13]. In our immunohistochemical study, Npas4 cells were expressed in the nucleus of neurons in the hippocampi and adjacent neocortices of pilocarpine-induced epileptic rats, which is consistent with previous studies [15], [28]. As a transcription factor, Npas4 works to regulate the inhibitory synapse number through the transcriptional control of effector genes [14]. The transcription factor Npas4 is known to regulate the transcription of exon-I and -IV [25]. Therefore, Npas4 may participate in the pathogenesis of epilepsy.

Our study also showed that seizure attacks were inhibited after Npas4 knocked down using specific siRNA interference, which suggested that Npas4 inhibit seizures. After siRNA interference, Npas4 protein expression was successfully inhibited, which was confirmed by the significant decrease of Npas4 protein expression in the Lv-Npas4-sh group compared with those in the normal group, saline group, and empty vector group. At the same time, seizure activities were provoked in the Lv-Npas4-sh group, including decreased onset latencies and increased seizure frequency, duration and seizure attack rates compared with the other control groups. The results demonstrated that Npas4 inhibited seizure attacks. This result was consistent with previous hypotheses [32].

The specific mechanism by which Npas4 regulates seizures is still unclear. Npas4 has been demonstrated to critically regulate inhibitory synapse formation [15]. In addition, Npas4 plays a role in the development of inhibitory synapses by regulating the expression of activity-dependent genes, which in turn control the number of GABA-releasing synapses that form on excitatory neurons. In our study, double-label immunofluorescence staining showed that the Npas4 protein was co-expressed with MAP2, which had been shown to stabilize microtubules and was critical for neuritis outgrowth and synaptic plasticity [33]. However, Npas4 did not co-express with the astrocytes marker GFAP; therefore, the Npas4 protein was not expressed in neuroglia cells, and the role of Npas4 in epileptogenesis may result from axon guidance and synaptic plasticity rather than astrocyte activation. Neuronal activity may be defined as the spread of a signal in the form of chemical messengers from one neuron to another. This form of communication between neurons in the CNS is related to many important physiological activities, such as synaptic remodeling [6], memory formation [11], and neuronal survival [19]. Furthermore, it showed that Npas4 expression was increased in the study of learning and synaptic plasticity, which suggested that Npas4 be involved in aspects of synaptic plasticity [22]. In addition, Npas4 regulates the expression of Sox1. Npas4 knockout affects the function of Sox1, and synaptic activity increases in the rat olfactory cortex when Sox1 is knocked out, which causes spontaneous epileptiform activity and eventually leads to limbic system and spontaneous epileptiform discharges. Npas4 knockout rats demonstrate similar hyper-excitability and are more prone to seizures [18]. In addition, Yun et al. [36] discovered that the over-expression of Npas4 elevated CDK5 and p-SYN-1 expression levels and enhanced neurite outgrowth. SYN-1 has been characterized as one of the major phosphoproteins in nerve terminals and is thought to be involved in the regulation of neurotransmitter release [7], [29]. Phosphorylated and nonphosphorylated Syn-1 regulate neuronal development [3], [10]. Npas4 expression is reportedly rapidly activated by excitatory synaptic activity and turns on a program of gene expression that triggers the formation and/or maintenance of inhibitory synapses on excitatory neurons.[15] from Michael E. Greenberg. “Activity- dependent regulation of inhibitory synapse development by Npas4”, Nature, 10/30/2008. Thus, the inhibition of Npas4 expression using siRNA interference in the pilocarpine-induced epileptic rats reduces the inhibitory neurotransmitter release, resulting in increased susceptibility to seizures. Lin et al [15] reported that excitatory synaptic activity induced Npas4 in a Ca^2+^-dependent manner and that the level of Npas4 determined the number of functional GABAergic synapses by controlling a program of activity-dependent gene expression. The mechanisms of Npas4 responsible for protecting against epilepsy may regulate GABAergic synapse development through the transcriptional regulation of BDNF [24]. Therefore, elevated Npas4 expression may demonstrate an inhibitory effect in the acute phase. After knock down of Npas4, protective elements were weakened, the progenesis of GABA-mediated inhibition neurons decreased, and the pilocarpine-induced seizures were more severe.

In conclusion, our study first demonstrated the dynamic expression of Npas4 protein in a pilocarpine-induced epileptic rat model. Additionally, we found that Npas4 inhibited seizures. Because of the ethical and experimental conditions, we have not detected the expression of Npas4 in the brain tissue of patients with epilepsy. Additional research should investigate how Npas4 regulates the GABAergic synapse to inhibit epilepsy.
